# Currents Nutritional Practices of Nutritionists in the Management of Type 2 Diabetes Patients at Public Health Centres in Padang, Indonesia

**DOI:** 10.3390/nu13061975

**Published:** 2021-06-08

**Authors:** Ice Yolanda Puri, Barakatun-Nisak Mohd Yusof, Zalina Abu Zaid, Amin Ismail, Hasnah Haron, Nur Indrawaty Lipoeto

**Affiliations:** 1 Department of Dietetics, Faculty of Medicine and Health Sciences, Universiti Putra Malaysia, Serdang 43400, Malaysia; gs51916@student.upm.edu.my (I.Y.P.); zalina@upm.edu.my (Z.A.Z.); 2 Department of Nutrition, Faculty of Public Health, Andalas University, Padang, West Sumatera 25175, Indonesia; 3 Department of Nutrition, Faculty of Medicine and Health Sciences, Universiti Putra Malaysia, Serdang 43400, Malaysia; aminis@upm.edu.my; 4 Nutritional Science Programme, Centre of Healthy Ageing and Wellness, Faculty of Health Sciences, Universiti Kebangsaan Malaysia, Kuala Lumpur 50300, Malaysia; hasnahharon@ukm.edu.my; 5 Department of Nutrition Sciences, Faculty of Medicine, Andalas University, Padang, West Sumatera 25127, Indonesia; indralipoeto@med.unand.ac.id

**Keywords:** current nutritional practice, nutritionists, type 2 diabetes, nutrition education module

## Abstract

(1) Background: The interest in nutrition practices and education is slowly gaining traction among Indonesian nutritionists. However, there is a lack of local studies that evaluate nutritional practices, especially in the management of type 2 diabetes (T2DM). This cross-sectional study aimed to determine the nutritional practices among nutritionists and the adequacy of the current practices in the management of Type 2 Diabetes Mellitus (T2DM) patients at the Public Health Clinic in Padang (PHC), Indonesia. (2) Methods: An online survey form was distributed to all the nutritionists (n = 50) involved in the management of T2DM patients in their daily practices at the PHC. Socio-demographic characteristics, the current practice of T2DM, the need for DM nutrition education, and an evaluation questionnaire on the Indonesian Non-Communicable Diseases guideline and the Public Health Centre guideline were captured in the survey. (3) Result: A total of 48 completed survey forms were received, providing a response rate of 96% from the recruited nutritionists. One-third (37.5%) of the respondents counselled between one and ten patients per day. Nearly half (41.7%) conducted a monthly follow-up session for the patients at their respective PHC in the previous three months. Each nutritionist educated five to ten T2DM patients. The most common nutrition education topics delivered included appropriate menus (89.6%) as well as the etiology and symptoms of T2DM (85.5%). Almost all the nutritionists (93.8%) used leaflets and about 35.4% used poster education. Around 70.8% of counseling sessions lasted 30 min and two-thirds (66.7%) of the sessions included nutrition education. Based on the results, about half (52.1%) of them claimed that T2DM patients were reluctant to attend individual nutrition education. One-fifth of them (20.8%) claimed that it was because the T2DM patients were not interested in the tool kits and materials used. (4) Conclusions: T2DM patients are reluctant to attend individual nutrition education due to uninteresting tool kits and materials.

## 1. Introduction

Globally, diabetes mellitus (DM) is a significant public health concern. The prevalence of DM is increasing rapidly in developing countries, especially in the Southeast Asian region [1]. Indonesia is not spared of the DM epidemic with a high prevalence of about 10.9%, whereby 95% of the diagnosed cases are Type 2 DM (T2DM). Padang is the largest city in West Sumatra and one of the most populous cities in Indonesia. In recent decades, the city has undergone rapid economic development and urbanisation, leading to a nutritional transition among its citizens. While the overall prevalence of DM in the province of West Sumatra is rather low at 1.8% [2], the prevalence in Padang City is relatively high, ranging from 6.4 to 10.6% based on the records the Padang City Health Centres [3]. The higher than average prevalence rate of DM in Padang city calls for urgent attention towards the problem to develop the appropiate approach in the management of diabetes at the Public Health Centre (PHC).

Nutritional therapy and education are integral components of T2DM management [4]. The provision of nutrition education has been shown to improve glycaemic control, body weight, and cardiovascular risk factors in patients with T2DM [5]. Furthermore, more significant weight reduction is observed when the nutrition education is delivered by a nutrition expert, a dietitian, or a nutritionist, as compared to other healthcare personnel [6]. Moreover, patients with T2DM showed an improvement in the knowledge about food choices and dietary practices after education sessions [7]. The improvement in knowledge and attitude is necessary to facilitate lifestyle modification to ensure sustainable and positive diabetes-related outcomes.

To date, many studies have reported on the roles and practices of dietitians or nutritionists in the management of diabetes [8,9,10]. Generally, all studies agreed that nutrition counselling is crucial in modifying eating habits, nutritional knowledge, and food preparation skills among individuals with T2DM [8,9,10]. However, there is a variation in the underlying adequacy and characteristics of nutrition services. In Australia, the dietitians managed 1–18 diabetes cases per week at 30–90 min for each case [8]. While the dietitians arrange at least four visits yearly, they received only two visits per annum on average. A lack of referrals and the prevalence of some physicians to deliver the nutrition counselling themselves can be the reason behind the low utilization rate of Australian dietitian services in the primary care setting, especially in remote areas. In South Africa, although a few diabetes education programmes and onsite interventions are available for diabetic patients, none of these materials are direcly related to the self-management and education of DM. There is also a lack of a structured nutrition component in the current programmes [9]. In comparison, dietitians New Zealand are involved in providing nutrition care to patients as well as enhancing the skills of other health care professionals in delivering nutritional knowledge and health promotion to their local population [11].

Locally, in Indonesia, a dietitian is a healthcare professional who provides clinical service at the tertiary care levels as compared to a nutritionist who is responsible for managing nutritional issues at the primary care and community levels, including at the PHC [12]. In a previous study, dietitians were also to play an essential role in providing nutritional care for wound management among T2DM patients with foot ulcers [13,14]. Nonetheless, little is known about the diabetes nutritional practices provided by nutritionists in the primary care setting. Moreover, the adequacy of nutrition services is also unknown, as well as the influence of nutrition education tools on the effectiveness of the education. The limitation of this study is that the study is only among nutritionists’ perspectives. Therefore, we need to continue the study to incorporate T2DM patients’ perspectives.

Recently, the interest in nutrition practices and education provided by Indonesian nutritionists is emerging. Although the Indonesian Department of Health has produced clinical practices guidelines to guide the medical treatment and management of T2DM, information about nutrition education remains minimal [9,10]. Therefore, this study aimed to determine the nutrition practices of among the nutritionists and the adequacy of the current practices in the management of T2DM at the PHC. The findings would be beneficial to enhance the services provided by nutritionists in the management of T2DM in the public health setting.

## 2. Materials and Methods

### 2.1. Study Design, Sampling Method, and Subjects

This was a cross-sectional study using an online survey (Google Form) to assess the current nutrition practices of T2DM management among nutritionists working at the Public Health Centres (PHC) in Padang, West Sumatra, Indonesia. The study was approved by the institutional Ethics Committee for Research Involving Human Subjects at the study site (Faculty of Medicine, Andalas University; No. 423/KEP/FK/2019) and home (JKEUPM-2019-423). Written informed consent was obtained from all subjects before the study.

### 2.2. Sampling Method and Subjects

The study sampled all nutritionists working in 23 PHCs across all districts (n = 11) in Padang, West Sumatra, Indonesia. From the database, there were a total of 50 nutritionists working in PHC during the data collection period between August 2019 and September 2019. An online survey was distributed to all nutritionists and 48 of them returned the survey within the stipulated period, thus providing a response rate of 96%. Two nutritionists declined to participate due to other work commitments.

### 2.3. Questionnaire

The questionnaire was developed based on the guidelines of the Integrated Health Service and Promotion in the prevention of Non-Communicable Chronic Diseases [11]. It was modified to better identify the needs of the current nutrition practice in T2DM management [11]. This questionnaire consisted of 18 multiple-choice and open-ended questions. The 18 questions included socio-demographic characteristics (4 questions), current practices in the management of T2DM (7 questions), and provision and adequacy of nutrition education (6 questions). The questionnaire was pre-tested among nutritionists (n = 12) working in different districts outside of the Padang area.

### 2.4. Data Analysis

The survey responses were analysed using SPSS version 25 (SPSS, Chicago, IL, USA). Descriptive statistics were used to summarise the information about the respondents’ sociodemographic characteristics, current practices, nutrition education provision and adequacy of services.

## 3. Results

A majority of the respondents were female (95.8%) with an average age of 40 years old. Half of them obtained a diploma in nutrition (Table 1). More than half of them (62.5%) had been a nutritionist at the PHCs for more than five years (Table 1).

Table 2 shows the current nutrition practices of T2DM at the PHC. The majority of the respondents (41.7%) conducted nutrition education sessions every month. Only 22.9% of the sessions were for patients referred from a physician. However, two-thirds of the respondents (68.7%) reported that they saw less than ten patients per month in either individual (93.8%) or group sessions (93.8%). About 80% of the respondents spent 30–60 min with patients in each session. They relied mainly on the Indonesian Public Health Care guidelines (77.1%) [12] and the Balanced Nutrition Guide [13] as a reference for nutrition practices and education for the management of T2DM.

The five most common topics for nutrition education delivered by the respondents included menu plans (89.6%), the definition of diabetes (81.3%), symptoms and etiology (85.4%), use of food models to replicate real food (75.0%), and carbohydrate exchanges (72.9%) (Table 2). In-house leaflets were the most common nutrition education aid (93.8%) used during the session. However, these leaflets were not standardised at each of the PHCs (Table 2).

Table 3 shows the perception of respondents about the adequacy of the current nutrition services available for patients with T2DM at PHCs. Most respondents perceived that the patients had a moderate level of knowledge (56.3%) but a positive attitude (95.8%) after receiving nutrition education sessions. More than half (54.2%) felt that the blood glucose levels of the patients reduced after the session. However, one-quarter (25%) of the respondents were unsure of the impact of nutrition education on blood glucose levels.

In general, the respondents (70.8%) felt that the nutrition education session was good. However, more than half (52.1%) of them perceived that many patients remained reluctant to attend the nutrition education session. Another (42.1%) of respondents were unsure if the nutrition services were inadequate. As high as three-quarters of the respondents (75%) expressed their concern that educational aid was not attractive (62.5%) or up to date (12.5%) (Table 3).

## 4. Discussion

The study presents the baseline characteristics of nutritionists, their current practices, and their perceptions about the adequacy of the nutritional services in the management of T2DM at PHCs in Padang, Indonesia. In Indonesia, the minimum requirement to be a nutritionist is a diploma, thus making the qualification lower compared to other developed countries. Ideally, dieticians and nutritionists should have minimum qualification of a Bachelor of Dietetics from an accredited university. In Zimbabwe, dietitians and nutritionists need to fulfill 1200 clinical hours and be registered with the Allied Health Practitioners Council of Zimbabwe before they can practice in the clinical setting. Furthermore, they should constantly keep themselves updated with the latest developments in their field [10]. The New Zealand (NZ) Primary Healthcare Centres found similar barriers in the patients’ management of DM even though NZ requires a dietitian to have specific qualification with varying degrees of experience and be registered with the Dietitians Board of NZ [11,18]. Similarly, the accredited Practicing Dietitians (APDs) in Australia are also required to be an accredited graduate certified in diabetes education and care and they must have undertaken a minimum of 1000 h of practice in diabetes education [19]. 

In this study, even though the majority of the respondents scheduled at least 12 visits annually for diabetes-related nutrition education, they only managed to see less than ten T2DM patients per month. The reported numbers were relatively low compared to Australia where the dieticians see at least 1–18 diabetic cases per week, or 4–72 cases per month, with patients scheduling two visits per year [8]. A cross-sectional study in 2019 reported that the number of T2DM patients that received nutrition education based on the attendance records from the previous year ranged from 4 to 19 at six selected clinics across nine health districts. The average number of T2DM patients seen by the diabetes educator were eight patients per week, or 32 cases per month [10]. The low number of patients could be related to the lack of interest of their preference to consult a doctor. A narrative review about the challenges in providing an effective individual nutrition education emphasized that the duration, the frequency, the aims, and the feedback to the patients should be given due consideration to enhance the outcomes of the programme, especially on the adherence to nutrition education. Additionally, non-effective nutrition education and confusion when answering patients’ questions might offset the benefit of nutrition education [20]. Similarly, other pertinent issues in nutrition education in NZ included the limited time available for the session and a lack of belief in the effectiveness of the education [11]. In China, registered dietitians and other multidisciplinary healthcare professional reported a lack of consistency in the research evidence on dietary barriers and the high cost of health food [21]. Apart from that, healthcare professionals struggle to cater to an increasing number of T2DM patients, especially if they have to fulfill other duties [22]. In reality, many health professionals in care practices are very busy managing various clinical. One of the solutions to overcome the time constraint is by reducing the duration of the health education or by supplementing it with other materials such as leaflets or booklets [23].

From the survey, the dieticians felt that some of the T2DM patients could be reluctant in attending individual nutrition education sessions due to uninteresting to tool kits and materials. In addition, some of the individuals with T2DM claimed that there was no extra benefit in seeing a dietitian as they already know what to do and they could obtain all the necessary information on the Internet. Furthermore, some individuals with T2DM were completely reluctant to modify their diet and to visit a dietitian. One of the common reasons individuals with T2DM fail to visit a dietitian was due to the lack of referrals from general practitioners. Very often, the general practitioners do not emphasise the importance of seeing a dietitian to the patient [24]. In China, a diabetes education programme invited 2812 individuals with T2DM. However, many participants declined (n = 1004), citing the reasons of frequent travel, being busy with work, lack of interest, or being uncontactable by phone [25]. The same study identified that individuals with T2DM showed a moderate level of knowledge, but had a positive response, and an improvement in blood glucose level after nutrition education sessions. However, in terms of the perception of the adequacy of nutritional services, many agreed that the inadequacy could be associated with the reluctance of individuals with T2DM to attend a nutrition education session. Dietitians and nutritionists have the responsibility to improve patients’ knowledge and behavior in terms of adherence, blood glucose monitoring, healthy diet, and physical activity. They should stress the importance of diabetes education and routine medical consultation because effective dietary counselling can lead to healthy dietary habits that subsequently improve the quality of life [18,20,26].

In term of duration per session, nutritionists in the survey held about 30 to 60 min visits. Nutrition education sessions in Zimbabwe, Australia, and New Zealand were delivered as both individual and group sessions. Similar to Indonesia, dietitians in Zimbabwe needed 30 to 60 min to conduct one-on-one or group nutrition education counselling. Meanwhile, dietitians in Australia and NZ generally spend 30 to 90 min for one-on-one, routine medical consultations and patient-centered diabetes care [8,9,10,11]. Accredited Practicing Dietitians (APDs) invited individuals with T2DM at least twice a week to nutrition education sessions via an advertisement posted in their electronic newsletter [8]. In this survey, the results indicated that the current nutrition education session was adequate. Nevertheless, there are still various obstacles in nutrition education delivery in the PHC, especially in terms of the reasons behind the reluctance of individuals with T2DM in attending individual nutrition education sessions. Even though nutritionists and healthcare professionals have their respective responsibilities and expertise, they must work together as a team. However, there is often an insufficient number of nutritionists to cater to the number of patients. Furthermore, nutritionists with high qualifications such as bachelor degree holders need to fulfill other administrative and financial commitments in the department, thus further reducing their availability to provide diabetes nutrition education to individuals with T2DM.

Currently, about three-quarters of the nutritionists use the Indonesia Public Health Care guidelines [16] and the Balanced Nutrition Guide [17] to guide their daily practices. The Balanced Nutrition Guide contains ten messages, such as accepting a variety of foods, consuming vegetables and fruits, high protein intake, intake with a variety of carbohydrate sources, limited intakes of food high in sugar and fat, routine breakfast consumption, regular exercises, frequent water intake, washing hands, and reading the food labeling. However this guide targets the general healthy population rather than the specific needs of individuals with T2DM. Thus, such a general healthy eating guide may limit the food recommendation for individuals with T2DM, escpecially taking into consideration the varying effect of high sugar food, and the diet pattern (time, amount, and type). Although the Indonesian Department of Health has produced a clinical practices guideline to guide medical treatment and management of T2DM, only a small proportion of the nutritionists (15%) used them as a guide in delivering nutrition education. This could be attributed to the minimal information on nutrition education in the T2DM clinical practice guidelines [9,10]. In diabetes nutrition, the content of nutrition education should encompass an overview of T2DM management such as glycaemic target, complications, and the importance of lifestyle modifications [21,27,28]. All of these aspects are among the top five most common topics of education covered by 80%–85% of nutritionists. Furthermore, the nutritionists also delivered modifications of lifestyle practices that are specific to diabetes [21,28].

Additionally, in this survey, it was found that nutritionists prefer to used leaflets and posters when they delivered nutrition education topics such as menu plans, food models, carbohydrate exchanges, high sugar foods, healthy eating, and plate methods. In other countries, such as Australia and New Zealand, the structured DM education program includes the understanding DM and medication, the promotion of a healthy lifestyle, and ways to avoid complications. Nutrition-wise, patients are educated about how to establish healthy meal plans and preparations, as well as how to achieve healthy eating at a low or no extra cost budgets. Similarly, in Zimbabwe, specific topics are added in terms of counting carbohydrate grams using online resources and the use of modified plate method approaches. The addition of these topics leads to an improvement in the nutritional status, blood pressure, HbA1c, fasting blood glucose, and dietary intake of the participating patients [8,9,10,11]. Even though nutrition education in Padang, Indonesia strives to achive the same outcomes, unfortunately, most of the nutrionists here are not familiar with HbA1c. National Basic Health Research did not provide HbA1c, the number of individuals with T2DM seeing the nutritionists, or visits to inidividual nutrition education sessions. In contrast, we could find the data following the PHC anuual report. The National Basic Registry of DM does not capture the HbA1c levels among T2DM patients. There are also no records of patients who attend nutrion education sessions provided by the nutritionists. Fortunately, the data is available in the annual PHC reports. 

In addition, the study reported that the plate method (isi piringku) is commonly practised by nutritionists. In contrast, Zimbabwe followed the South African Food-Based Dietary Guidelines. Individuals with T2DM learned about the portion sizing for different food groups using the Zimbabwe hand jive images. Furthermore, based on the guidelines, T2DM patients should perform 150 min of moderate-intensity physical activity, as well as resistance activities three or more times a week. Housework is also considered as one such activity. Another qualitative study on the current practices of DM management at PHC in NZ conducted semi-structured, face-to-face interviews with dietitians. The healthcare professional team had access to the electronic health records of the patients. Thus, it facilitated the delivery of nutrition education to the patients in the local community either via individual or group session [8,9,10,11,27].

Furthermore, the survey results showed that individuals with T2DM had a moderate level of knowledge but showed a positive response, and improvement in blood glucose level after nutrition education sessions. This is indicative of the critical role of nutritionists improving patient’s knowledge and adherence to the self-monitoring of blood glucose [18,20]. According to the findings, many nutritionists attributed the lack of adequacy of the nutrition education to the lack of patients’ interest in attending the session and outdated pedagogy. Therefore, it is imperative to provide a structured nutrition education module for individuals with T2DM. At present, almost all nutritionists relied on leaflets as an educational aid. However, studies have shown the lack of efficacy of leaflets. For example, the Australia General Practice Management (GPM) emphasised that simple health education such as leaflets was unlikely to modify health behaviors [18]. On the other hand, the use of colourful booklets, a plate during regular lectures, and group visits are more beneficial in improving the overall glycaemic control in individuals with T2DM [25]. None of the previous studies in Padang and Indonesia evaluated the reason why T2DM patients are reluctant to attend individual nutrition education. Twenty-two individuals with T2DM in the intervention group were given NCP treatment, and 22 subjects in the control group received standard service of chronic diseases service program. There were no significant differences in the fasting blood glucose before treatment. However, significant differences were detected in the knowledge, attitudes, and diet compliance after intervention. In the local setting in Padang showed no significant improvement in knowledge, attitude, or behavior with T2DM after the application of the Interactive Community Based Approach (CBIA) for knowledge, attitude, and behavior, and the KIA model [29,30,31].

## 5. Conclusions

In summary, half of the nutritionists in this survey counselled T2DM patients every month. Most of them relied on the Balanced Nutrition Guide that is not tailored for individuals with T2DM. The outdated education tools could be attributing to the low interest and reluctance among the patients in attending the nutrition education session. The outcomes emphasised the need for revamped education tools that are effective among the local T2DM population in Indonesia. Further studies should evaluate the potential reasons behind the lack of interest in attending the nutrition education sessions among patients with T2DM so that corrective strategies can be put in place.

## Figures and Tables

**Table 1:** Sociodemographic characteristics of the nutritionists (n = 48).

Items	n	%
**Sex**		
Male	2	4.2
Female	46	95.8
**Age**		
**Mean (SD)**	40.04 (±6.0)	
Mean age ≤ 40 years old	26	54.2
Mean age > 40 years old	22	45.8
**Highest Education Level**		
Diploma	24	50.0
Bachelor	23	47.9
Masters	1	2.1
**Work Experience**		
≤5 years	18	37.5
>5 years	30	62.5

**Table 2:** Current nutrition practice of the nutritionists at public health clinics (n = 48).

Nutrition Practices Component	n	%
**Frequency of Visits for Nutrition Education at *Puskesmas* (Public Health Centres)**		
Once a month	20	41.7
Twice a month	9	18.8
Once a week	8	16.7
Others (Based on physicians referral)	11	22.9
**Number of patients seen monthly**		
<10	33	68.7
11–19	12	25.0
>20	3	6.3
**Delivery of Nutrition Education**		
Group education	45	93.8
Individual nutrition education	45	93.8
Others (home visit and joining with the nursing station)	3	6.2
**Duration per session**		
<30 min	10	20.8
30–60 min	38	79.2
>60 min	0	0
**Guide and reference for conducting nutrition education**		
Non-Communicable Diseases guidelines [15]	7	14.6
Indonesian Public Health Care guidelines [16]	37	77.1
Balance Nutrition Guide (*Pedoman Gizi Seimbang)* [17]	34	70.8
**The Most commonly covered nutrition education topics**		
Menu Plan	43	89.6
Symptom and etiology	41	85.4
Pathophysiology of diabetes	39	81.3
Food Model tool kits	36	75.0
Carbohydrate exchange	35	72.9
High sugar foods	33	68.8
Balanced Nutrition Guide (*Pedoman Gizi Seimbang*)	31	64.6
Plated method (*Isi piringku*)	30	62.5
Provide prescription on sugar consumption	29	60.4
Provide macronutrients	27	56.3
Carbohydrate counting	25	52.1
Provide a proper cooking method for the menu plan	21	43.8
Provide prescription on fibre	17	35.4
Read label	15	31.3
Use the Indonesian food pyramid	12	25.0
Exercises	10	20.8
**Most commonly used nutrition education aid**		
Leaflet	45	93.8
Poster	17	35.4
Short Messages System and (What’s App)	3	6.3

**Table 3:** Perception of nutritionists (n = 48) about the adequacy of the current nutrition services available for people with T2DM at the public health clinic.

Items	n	%
**Patients’ knowledge after the nutrition education session**		
High	3	6.3
Moderate	27	56.3
Low	18	37.5
**Patients’ attitude after the nutrition education session**		
Positive	46	95.8
Negative	1	2.1
Not sure	1	2.1
**Patients’ improvement in blood glucose**		
Reduced	26	54.2
Increased	6	12.5
No change	4	8.3
Not sure	12	25
**Adequacy of the current nutrition education session**		
Very Good	6	12.5
Good	34	70.8
Moderate	8	16.7
**Reasons of nutrition services inadequacy**		
Patients reluctant to attend a nutrition education session	25	52.1
Patients prefer going to the hospital and/or medical doctor	14	22.9
Lack of budget to improve nutrition service	5	10.4
Nutrition services and activity is not interesting	4	8.3
Not sure	20	41.7
**Perception about the current nutrition education aid**		
Not attractive	30	62.5
Not up to date	6	12.5
Not sure	26	54.2

## Data Availability

No supplement data.
